# Uncovering correlated variability in epigenomic datasets using the Karhunen-Loeve transform

**DOI:** 10.1186/s13040-015-0051-7

**Published:** 2015-07-01

**Authors:** Pedro Madrigal, Paweł Krajewski

**Affiliations:** 1 Department of Biometry and Bioinformatics, Institute of Plant Genetics of the Polish Academy of Sciences, Strzeszyńska 34, Poznań, 60-479 Poland; 2 Present address: Wellcome Trust-MRC Cambridge Stem Cell Institute, Anne McLaren Laboratory for Regenerative Medicine, Department of Surgery, University of Cambridge, West Forvie Building, Forvie Site, Robinson Way, Cambridge, CB2 0SZ UK; 3 Present address: Wellcome Trust Sanger Institute, Wellcome Trust Genome Campus, Hinxton, Cambridge, CB10 1SA UK

**Keywords:** Histone modifications, ChIP-seq, Functional data analysis, Stem cells, H1, Roadmap Epigenomics Consortium, H3K4me3, H3K36me3, H3K9ac, H2A.Z

## Abstract

**Background:**

Larger variation exists in epigenomes than in genomes, as a single genome shapes the identity of multiple cell types. With the advent of next-generation sequencing, one of the key problems in computational epigenomics is the poor understanding of correlations and quantitative differences between large scale data sets.

**Results:**

Here we bring to genomics a scenario of functional principal component analysis, a finite Karhunen-Loève transform, and explicitly decompose the variation in the coverage profiles of 27 chromatin mark ChIP-seq datasets at transcription start sites for H1, one of the most used human embryonic stem cell lines. Using this approach we identify positive correlations between H3K4me3 and H3K36me3, as well as between H3K9ac and H3K36me3, so far undetected by the most commonly used Pearson correlation between read enrichment coverages. We uncover highly negative correlations between H2A.Z, H3K4me3, and several histone acetylation marks, but these occur only between principal components of first and second order. We also demonstrate that levels of gene expression correlate significantly with scores of components of order higher than one, demonstrating that transcriptional regulation by histone marks escapes simple one-to-one relationships. This correlations were higher in significance and magnitude in protein coding genes than in non-coding RNAs.

**Conclusions:**

In summary, we present a methodology to explore and uncover novel patterns of epigenomic variability and covariability in genomic data sets by using a functional eigenvalue decomposition of genomic data. R code is available at: http://github.com/pmb59/KLTepigenome.

**Electronic supplementary material:**

The online version of this article (doi:10.1186/s13040-015-0051-7) contains supplementary material, which is available to authorized users.

## Background

Mechanisms orchestrating fundamental biological processes, such as cellular division and differentiation, are greatly affected by epigenetic regulation of gene expression [1]. By definition, epigenetics is explained by DNA methylation status and modifications of histone proteins within the nucleosomes, allowing an inherited phenotype not linked to changes of DNA sequence. The epigenetic landscape is constituted by a wide and complex array of combinations of histone modifications, chromatin regulators, and non-coding RNAs acting in a coordinated way, resulting in temporally consistent, cell-, and tissue-specific gene expression [2]. Understanding these dynamic processes has become much easier with recent advances in high-throughput sequencing [3]. Despite inherent biases [4], the pertinence of chromatin immunoprecipitation followed by high-throughput sequencing (ChIP-seq) [5, 6], DNase-seq [7], FAIRE-seq [8], ChIP-exo/ChIP-nexus [9, 10], and ATAC-seq [11], among others, to unravelling the location of transcription factor (TF) binding events, nucleosomes, histone modifications and regions of accessible chromatin is widely recognized [12–14].

One of the key problems in computational epigenomics is the poor understanding of associations between epigenetic signals. Though individual correlations of read-enrichment or co-localization of chromatin marks have been documented in various contexts, it is frequently unknown whether a particular epigenetic signal is governed by similar patterns on other levels of variability beyond the primary one [15]. More than 100 distinct histone post-translational modifications have been identified thus far, and likely many more have not been uncovered yet [16]. As the biological functions of most of these histone modifications and histone variants are largely unknown [17], and context-dependent, in practice researchers limit their inquiries to a reduced subset of them. For instance, a set of six key histone modifications (H3K4me1, H3K4me3, H3K9ac, H3K9me3, H3K27me3, H3K36me3) was initially defined for human epigenome analysis by the Roadmap Epigenomics Consortium to be most informative, and having relatively well-known function and antibodies of reasonable quality [3]. Finally only five of them constituted the core set, with H3K9ac (and H3K27ac) included only in selected epigenomes [2]. Many other epigenetic marks, along with their aberrant levels that potentially trigger or anticipate disease states, are expected to be identified in the foreseeable future [18], with the International Human Epigenome Consortium aiming to go far beyond the Roadmap Epigenomics Consortium, up to more than 1000 human reference epigenomes [3]. Novel analytical methods will facilitate the transition from the analysis of few to multiple epigenetic marks.

Variability is normally observed in next generation sequencing (NGS) data as a result of technical biases in library preparation [19], batch effects [20], or sequencing errors [21]. Undesirable sources of variation can be partly controlled at the experimental design step by assigning an appropriate number of biological/technical replicates, and randomizing samples across library preparation batches and lanes. The variation that is of interest is observed as a consequence of experimental treatments, genotypes, cell types, cell cycle, or between single cells [22]. Remarkably: (i) a large diversity and variation exist in epigenomes both across cell types and individuals [23]; for example up to 95-fold variation between reference epigenomes has been observed in H3K4me3 signal close to TSSs in humans [24]; (ii) chromatin mark features such as length or read-enrichment shape have proven to be relevant for gene expression regulation [25, 26]; (iii) chromatin marks can capture differences that are not reflected in either methylation or DNA accessibility [2]; and (iv) these marks may have additional practical applications, as shown recently by the histone variant H2A.X, which variability can be used as an indicator of quality in mouse induced pluripotent stem cells [27]. Therefore, statistical measures of variation and genome-scale integrative analyses of multidimensional and multimodal data are necessary [28, 29]. To this end, chromatin states and novel functional elements of the histone code [30] have been inferred and annotated, respectively, from histone modifications acting in a combinatorial fashion by using multivariate hidden Markov models [2, 31, 32], dynamic Bayesian networks [33, 34], or unsupervised machine learning [35]. Missing marks imputed from existing data have been used to expand the number of chromatin state annotations [24]. However, complementary to these genome segmentation approaches that divide the genome according to histone combinations, quantitative rather than qualitative (segmentation-based) analyses are necessary, as demonstrated, e.g., to reveal patterns produced by TF-DNA binding events [36, 37], or to infer novel interactions in histone networking [38, 39]. Thus, both large-scale datasets and novel computational approaches are fundamental to decipher the histone code that controls the functionality of chromatin by modulating nucleosomal structure [17].

Herein we describe a dimensionality reduction approach based on functional principal component analysis (FPCA), a finite realization of the Karhunen-Loève transform, to quantify the covariability existing between ChIP-seq datasets with respect to defined loci or regions of interest. This allows to investigate the dominant modes of variation in the data using the eigenfunctions of the covariance function. A region-of-interest based analysis increases the interpretability and statistical power when searching for differences in epigenomic data [40, 41]. Multivariate PCA has been used to identify distinct chromatin states [42, 43], but to the best of our knowledge there has been no attempt to decompose chromatin marks into principal components (linear combinations of the original variables) to study their correlation. FPCA has been proven to adequately detect information from shapes (of the curves) that cannot be identified by traditional multivariate statistics [44], and computationally it allows representing data in large genomic regions by a reduced set of coefficients. We demonstrate the advantages of FPCA over the computation of ordinary (Pearson) correlation coefficients of genomic enrichment profiles. These are now commonly used to identify correlated genomic features (e.g., within the proximal promoter regions in ChIP-seq data sets [45]). However, by definition they are able to identify correlated signals only if they occur in the same genomic intervals. We also demonstrate the applicability of FPCA for studying quantitative relationships between principal components of histone modifications and gene expression levels, which is otherwise limited due to the unidimensional analysis of read-enrichment commonly used for ChIP-seq data. Overall, our method allows a more in-depth analysis of the correlations between epigenetic changes than conventional methods.

As a case study we use ChIP-seq data from the Roadmap Epigenomics Consortium [2, 46]. Our results reveal the decomposition of NGS coverage profiles in the functional space of their principal components using transcription start sites (TSSs) as reference landmarks. We found previously unknown differences between H3K4me3 and H3K9ac uncovered by the second principal component, and linked to opposed changes in gene expression. Similarly, we detected significant correlations between H3K36me3 and other chomatin marks such as H3K4me3 and H3K9ac in the first component, which cannot be revealed using correlation coefficients based on the ordinary coverage correlation. Highly negative correlated signatures were found between H2A.Z, H3K4me3, and several histone acetylation marks, but only between components of order 1 and 2. We also find a significant correlation between FPC scores and gene expression, not only at first but also at higher order components. The results strongly suggest that higher order principal components are chromatin mark features that have a direct impact on gene expression, and that correlate with the components of other chromatin marks, thus shedding light into the complexity of the cross-talk between these histone-code key players.

## Methodology

### Functional data analysis

Functional data analysis encompasses many methodologies for statistical analysis of data coming from measurements concerning continuous phenomena, e.g., curves or surfaces [47]. The continuum is usually time, but it might be also spatial location or another coordinate system. Below we briefly review the foundations of functional principal component analysis.

### Karhunen-Loève transform in an infinite-dimensional space

By the Karhunen-Loève theorem [48], a function *x*(*t*), centered and square-integrable on an interval $[a,b] \subset \Re $, can be represented by the linear combination

(1) $$ x(t)=\sum\limits_{k=1}^{\infty} \eta_{k} \xi_{k}(t),   $$

where *ξ*_1_(*t*), *ξ*_2_(*t*), … are orthonormal deterministic eigenfunctions, and the coefficients *η*_1_,*η*_2_,… are uncorrelated random variables defined as

(2) $$ \eta_{k}={\int_{a}^{b}} x(t) \xi_{k}(t) dt, \hspace{5 mm} k=1,2,\ldots.   $$

The covariance function of *x*(*t*) is of the form

(3) $$ v(s,t)=cov\left(x(s),x(t) \right)=\sum\limits_{k=1}^{\infty} \lambda_{k} \xi_{k}(s) \xi_{k}(t), \qquad s,t\in [a,b],   $$

where *λ*_*k*_=*var*(*η*_*k*_) are the eigenvalues corresponding to the *k*-th eigenfunction. The first eigenfunction *ξ*_1_(*t*) represents the dominant mode of variation of *x*(*t*), as it maximizes the variance of *η*:

(4) $$ var(\eta)=var\left[{\int_{a}^{b}} x(t) \xi(t) dt \right] = {\int_{a}^{b}} {\int_{a}^{b}} \xi(s) v(s,t) \xi(t) ds dt.   $$

Thus, in a functional orthogonal space, the function *x*(*t*) in [*a*,*b*] is represented by the vector of coefficients *η*_1_,*η*_2_,…. By definition of *v*(*s*,*t*), the eigenfunctions satisfy the equation

(5) $$ {\int_{a}^{b}} v(s,t) \xi_{k} (s) ds = \lambda_{k} \xi_{k}(t), \quad k=1,2,\ldots,   $$

with *λ*_1_≥*λ*_2_≥…. By solving equation (5) the functional components *ξ*_1_(*t*), *ξ*_2_(*t*), … can be found.

### Functional principal component analysis for genomic data

Let *L* be the number of genomic regions in which an NGS read coverage profile (obtained, e.g., from a ChIP-seq, RNA-seq, or methylation experiment) is observed. If the regions are chosen in such a way that they have some characteristic in common, e.g., they all are TSSs, exons, CpG islands, etc., a natural question arises concerning the variability of the observations between the regions. We propose to analyse the data profiles by means of functional principal component analysis, a finite realization of the Karhunen-Loève theorem.

**Fig. 1:**
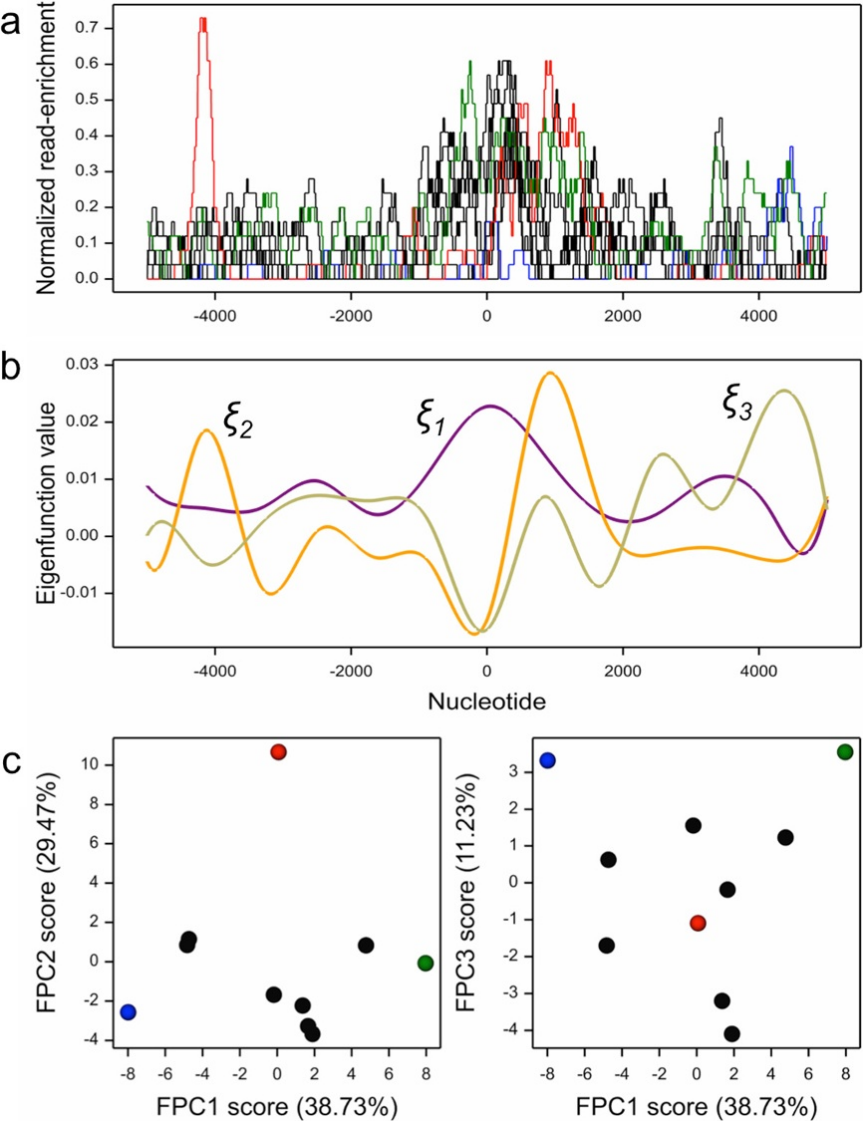
Basic procedure of the functional data analysis. (**a**) Ten observations of a function, (**b**) Estimated eigenfunctions; (**c**) Plot of data in the system of first, second, and third functional principal component scores. Because the eigenfunctions are orthonormal, each dominates others in some subintervals; these subintervals define the occurence of a signal corresponding to each eigenfunction. In (**a**) selected profiles are marked as green, red, and blue; their order in the subinterval around 0 dominated by first eigenfunction is the same as the order along the first FPCA axis in (**c**). The same holds approximately (due to loss of information in the two-dimensional graphs) for other functional principal component scores corresponding to eigenfunctions drawn in (**b**)

We denote the observed profile *i* (*i*=1,…,*L*) by *x*_*i*_(*t*). All data are then represented by the vector of functions $\bigl [ x_{1}(t), x_{2}(t), \ldots, x_{L}(t) \bigr ]^{T}$. Functional principal component analysis (FPCA) is accomplished numerically by the application of an expansion of the observed functions using a set of *K* basis functions *ϕ*_1_(*t*),*ϕ*_2_(*t*),…,*ϕ*_*K*_(*t*) [47]. We used a linear combination of B-spline functions defined by positioning equidistant knots along the peak regions. B-splines are the common approximation system for non-cyclical non-periodic data [49]. Thus, each profile *x*_*i*_(*t*) is first approximated by a linear combination

(6) $$ x_{i}(t)=\sum\limits_{k=1}^{K} c_{ik} \phi_{k}(t),   $$

where the coefficients *c*_*ik*_, *i*=1,…,*L*; *k*=1,…,*K* are estimated by least squares, but penalized residual sum of squares criterion can be used as well (for further details see [47]). However, when the sample curves are smooth and observed with error, least-square approximation in terms of B-spline basis functions is an appropriate solution for the problem of reconstructing their functional form [50]. Then, the eigenfunctions are estimated by solving (5) (which is numerically possible if we assume that also the eigenfunctions can be approximated by an expansion with respect to B-spline functions), and the *i*-th observed profile (in our case - genomic region) is given a representation in the functional space by a set coefficients *η*_*i*1_,*η*_*i*2_,… defined in (2), known as FPCA scores. By definition, first eigenfunction corresponds to maximum variability among the observed profiles. The vector of scores *η*_11_,*η*_21_,…,*η*_*L*1_ can be used to represent variation among regions with respect to coverage profile in one dimension, analogously to the first principal component in the multivariate analysis. The number of estimated eigenfunctions, *J*, can be at most equal to *K* [47]. Rules similar to the ordinary multivariate PCA can be applied to select *K*. A basic explanation of the steps involved in functional principal component analysis is given in Fig. 1.

In the rest of this paper, we will use FPCA not just to analyze variation within one data set (genomic track), but also to compare and link variation in pairs of data sets using common intervals [*a*,*b*]. For this we need to make the following observations:

- Large values of an eigenfunction define the regions marked by this eigenfunction as regions of large variation among the observed functions. For two data sets A and B, the correlation coefficient between two estimated eigenfunctions *ξ*_*A*_ in A and *ξ*_*B*_ in B, computed from their values in the interval [*a*,*b*], measures the co-occurence of variability in common regions between these two eigenfunctions.

- The FPCA scores related to an eigenfunction, computed for all observed functions, approximate the ordering of those functions in the genomic region(s) indicated by this eigenfunction as region(s) of large variation. The correlation coefficient between two sets of scores related to *ξ*_*A*_ and *ξ*_*B*_, measures the co-variation of the two sets of observed functions in the regions indicated as having large variation by the two eigenfunctions.

Thus, for each pair of functional principal components estimated in two data sets, we can compute two coefficients, one measuring the co-occurence of the regions of variation, and the other measuring the correlation of scores corresponding to this pair of components.

### ChIP-seq data normalization and statistical tests

Normalization of ChIP-seq data with respect to read number and read length was done using the module ‘normalize.bigwig.py’ in RSeQC [51]. Pearson correlation coefficients and tests for correlation between paired samples were computed using the statistical software R. P-values were corrected for multiple testing using the Bonferroni method where appropriate. Pearson correlation of read coverage was calculated by the UCSC ‘bigWigCorrelate’ function with the option ‘-restrict’ to limit the computation to TSS regions. We filtered out regions overlapping the 411 consensus artefact blacklisted regions [1], as not removing those can influence downstream results and correlation measures [52, 53]. GENCODE v10 annotation was used - ribosomal genes were excluded.

## Results and discussion

We downloaded ChIP-seq data sets corresponding to 27 different chromatin marks in H1 human embryonic stem cell (hESC) line (Additional file 1). We combined raw coverage profiles for a chromatin mark, normalized signal values across the genome (factor 100×10^6^), and applied functional principal component analysis in regions ± 5 kb around the TSSs to study the variation of deposition of one histone mark across the various genes in H1 cells, and to understand the correlation between different histone marks. Chromatin configuration in different regions of the genome, such as promoters, enhancers, and those of transcribed DNA, is defined by distinct histone modification patterns [32]. We chose TSSs as they are well annotated genomic features. The analysis was performed separately for protein-coding (pc) and non-coding (nc) RNAs (19762 and 31331 genes, respectively).

### H3K4 mono-, di- and tri-methylation correlated variation

**Fig. 2:**
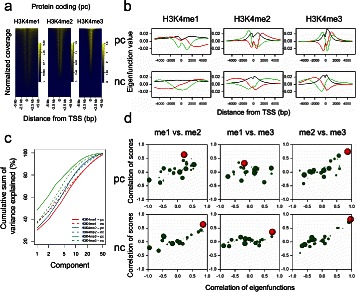
Results of functional principal component analysis for H3K4me1/me2/me3 data in H1 hESCs for TSSs of protein-coding and non-coding RNAs. (**a**) Heat maps of normalized coverage for H3K4me1, H3K4me2, and H3K4me3 ChIP-seq relative to TSSs of protein-coding genes. (**b**) First, second, and third eigenfunctions computed for different marks and for different groups of genes (black - FPC1, red - FPC2, green - FPC3). (**c**) Cumulative proportion of variance explained by 50 eigenfunctions for protein-coding and non-coding RNAs. X-axis is represented in log-scale. (**d**) Pairwise correlations between FPC scores computed for different histone marks (Y-axis) versus corresponding correlations between underlying eigenfunctions (X-axis). Results shown for 5 functional principal components. Symbol size proportional to the ranked product of variance explained by the components, red circle used for the correlation between components no. 1

We illustrate the method with the analysis of H3K4me1/me2/me3 data for pc and nc genes (Fig. 2). H3K4me2 and H3K4me3 are predominantly confined to narrow peaks, and many of these lie at the TSSs of annotated genes. On the contrary, H3K4me1 predominantly marks enhancer regions. Pearson correlation coefficients shown in Table 1 computed using full read-enrichment profiles in TSS regions report that the largest discrepancies exists between me1 and me3, the smallest between me2 and me3, and that such a comparison of profiles gives very similar results for pc and nc genes. To dissect the relationships between variations in different methylation marks we used FPCA (*K*=50 components), observing different configuration of eigenfunctions for pc and nc genes, especially for H3K4me1 close to TSSs (Fig. 2b). Cumulative profiles of variance explained by consecutive components show that variation in H3K4 tri-methylation marks, known to be found in TSSs of active genes, was more extensively captured than variation in mono- and di-methylation, suggesting that the deposition of H3K4me3 on TSSs follows less intricated read enrichment patterns (Fig. 2c). Within gene categories, the largest proportions of variance were captured for pc-associated H3K4me3, revealing that more differences exits in this chromatin mark for nc genes (profiles for nc not shown), but the same does not apply for H3K4me1/me2. This can be interpreted as the regulation of H3K4me3 on nc genes following a more complex regulation. We note that variation of H3K4 marks in nc genes took place in intervals wider than in pc genes, as seen from characteristics of variation defined by the eigenfunctions.

**Table 1:** Correlation characteristics of H3K4me1/me2/me3 data sets for protein-coding and non-coding genes based on read-enrichment profiles and on their functional principal components 1-5

Group of genes	Type of correlation	me1 vs. me2	me1 vs. me3	me2 vs. me3
Protein coding	Ordinary correlation	0.50	0.11	0.63
	Maximum correlation of *ξ*	0.55 (1,2)	0.72 (1,3)	0.86 (1,1)
	(component numbers),	0.27	0.14	0.75
	corresponding correlation of *η*			
	Maximum correlation of *η*	0.64 (1,1)	0.35 (3,3)	0.75 (1,1)
	(component numbers),	0.22	-0.37	0.86
	corresponding correlation of *ξ*			
	Significant pairwise correlations	590	203	646
Non-coding	Ordinary correlation	0.48	0.15	0.65
	Maximum correlation of *ξ*	0.86 (1,1)	0.76 (1,1)	0.96 (1,1)
	(component numbers),	0.64	0.36	0.82
	corresponding correlation of *η*			
	Maximum correlation of *η*	0.64 (1,1)	0.36 (1,1)	0.82 (1,1)
	(component numbers),	0.86	0.76	0.96
	corresponding correlation of *ξ*			
	Significant pairwise correlations	529	197	615

For further comparison of different H3K4 methylation marks we concentrated on first five principal components, and we computed pairwise correlations between eigenfunctions, and between principal component scores obtained for different marks (Fig. 2d). Highest correlation between eigenfunctions, that show the co-localization of variation in the same genomic intervals, was found between first components (*ξ*_1_,*ξ*_1_) for nc-related marks. A different situation was observed in pc genes, where the signal of largest variation in me1 co-occurs with signals of secondary (*ξ*_2_) or tertiary (*ξ*_3_) variation in, correspondingly, me2 and me3 marks (Table 1). In consequence, the correlations between the scores computed for the co-localizing components are approximately proportional to ordinary correlations measured on full profiles for nc genes, but are not for pc genes ($r_{\eta _{1},\eta _{2}}=0.27 < 0.50$, H3K4me1 vs. H3K4me2). Also, from Table 1 we can see that, for pc genes, between-scores correlation coefficients larger than those computed on the whole profiles can be found in components which eigenfunctions do not correlate ($r_{\xi _{3},\xi _{3}}=-0.37$, H3K4me1 vs. H3K4me3). Therefore, we interpret that the comparison of different H3K4 methylation marks using the ordinary correlation is informative for nc genes for which the largest correlations exist in colocalizing intervals characterized by the largest signal variation (first eigenfunctions), whereas it is not fully informative for pc genes, for which the largest correlations concern signals in intervals that are not the same for me1 on one hand, and for me2 and me3 on the other.

We then calculated pairwise correlations between principal component scores for all the components, detecting significant correlations (Bonferroni corrected *P*≤10^−6^) at higher-order components in the TSSs, that could not have been observed applying the ordinary correlation merely based on the read-enrichment in whole regions. These were higher in number for pc genes (Table 1), suggesting that H3K4 methylation cross-talk is more important in transcriptional regulation of pc genes. The distributions of the correlation coefficients between pc and nc genes were different for the H3K4me1-H3K4me2 comparison (Kolmogorov-Smirnov (K-S) test, *P*=0.00056), H3K4me2-H3K4me3 (KS test, *P*=0.026), and especially for H3K4me1-H3K4me3 (KS test, *P*=4.07×10^−6^). Overall, these analyses suggest that eigenfunction decomposition can be used to compare histone modification data at higher order. This example also shows how epigenomic variation between genes of distinct functional categories can be investigated using the proposed methodology.

### Comparison of ordinary coverage correlation versus FPCA-based correlations

**Fig. 3:**
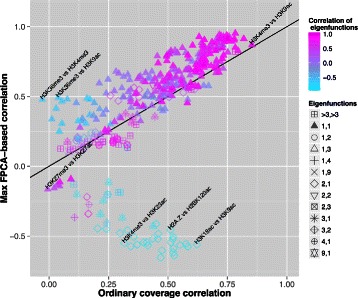
Comparison of ordinary coverage correlation versus FPCA-based correlations. Scatterplot of Pearson coverage-based correlation coefficients and maximum FPC-based Pearson correlation coefficients between the scores of H1 hESC line at TSSs. Color scale indicates the correlation of the corresponding underlying eigenfunctions. Shape code denotes the order of the pairs of eigenfunctions. Chromatin modifications discussed in the main text have been labeled. No change between the two measures is represented by the black line. All data can be found in the Additional file 1

We then computed pairwise Pearson correlations between the 27 data sets in TSSs associated to pc genes in order to compare them with the (absolute) maximum correlation values resulting from our FPCA-based approach. We found that highly correlated scores of chromatin marks were defined usually by the first components (*ξ*_1_,*ξ*_1_), which were also highly correlated (co-localized). On the contrary, low correlations corresponded often to interactions between components of order higher than 3 (Fig. 3; Additional file 1).

Despite the proportionality between the two measures, we found interesting cases of correlations detected only using our methodology, which corresponded in most cases to negative correlation between eigenfunctions. For instance, ordinary coverage correlation of H3K36me3 and H3K4me3 was *r*=−0.03, in agreement with a recent publication that found comparable results across 127 epigenomes [2]. However, we found the maximum correlation between the scores of their first components equal to $r_{\eta _{1},\eta _{1}}=0.48$ (*P*≤2.2×10^−16^, correlation test; Fig. 3). As both marks co-occur in active genes, H3K4me3 in promoter regions close to the TSS, and H3K36me3 in transcribed regions, it has been intriguing why correlation between these two histone modifications has been reported as approximately 0 so far. A similar scenario takes place between H3K36me3 and the active promoter mark H3K9ac ($r_{\eta _{1},\eta _{1}}=0.49$; Fig. 3), also not uncovered by the analysis of 127 epigenomes [2]. In both cases, the correlation between the eigenfunctions is negative ($r_{\xi _{1},\xi _{1}}=-0.61$), which indicates that regions of variability for the most important components do not co-localize. A possible interpretation could be that epigenetic marks on one nucleosome might affect (negatively) marks on neighboring nucleosomes. Opposed to this, as principal eigenfunctions have the same profile and both marks co-localize, high correlation between H3K9ac and H3K4me3 was detected by others [2], which is confirmed also in our analysis ($r_{\eta _{1},\eta _{1}}=0.87$, correlation of eigenfunctions $r_{\xi _{1},\xi _{1}}=0.99$). Overall, our method outperforms standard correlation measurements when the histone marks that are similarly regulated do not directly overlap.

Unlike the traditional measure of correlation, we found many negative FPC-based correlations which can be divided into three groups: low negative correlations, mostly in combinations of H3K27me3 and other marks, between their first co-localized components. For instance, we observed negative correlation between H3K27me3 and H3K27ac, which is expected as these marks are known to be mutually exclusive, but it was not detected by ordinary correlation; moderate negative correlation of a heterogeneous group of marks at high order components; and most surprinsingly, highly negative correlations between H2A.Z, H3K4me3, and several histone acetylation marks, these occurring only between principal components of first and second order, that do not co-localize. This suggests that differences between chromatin marks, even those highly correlated as acetylations, can be better detected by decomposition of the signal into principal components. Overall, this illustrates that the methodology we propose is able to uncover previously unknown relationships between histone modification ChIP-seq datasets.

### Eigenfunction decomposition of chromatin marks versus gene expression

**Fig. 4:**
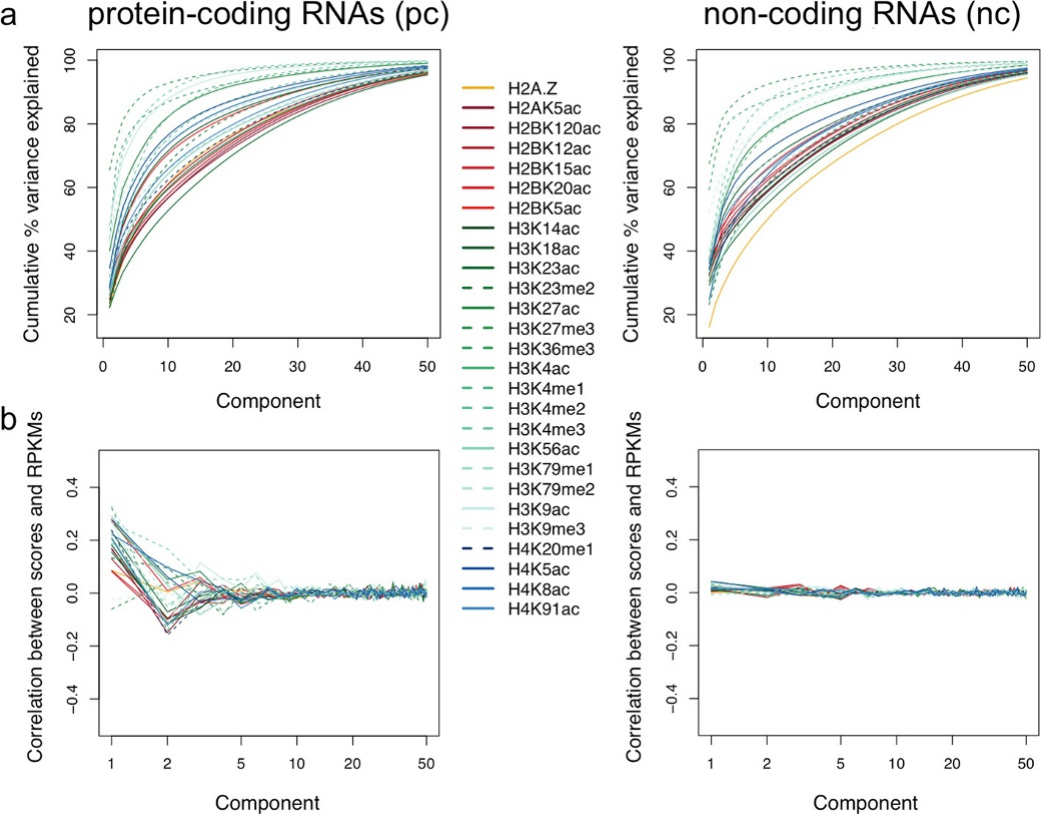
Correlation between chromatin marks and gene expression values. (**a**) Cumulative proportion of variance explained by 50 eigenfunctions for protein-coding and non-coding RNAs in 26 histone modification and one histone variant ChIP-seq data sets. Values correspond to FPCA analysis in regions ± 5 kb around the TSSs for H1 hESC. (**b**) Pearson correlation between RPKM gene expression values and functional principal scores for each chromatin mark. X-axis is represented in log-scale

The modification of chromatin structure brings unique transcriptional signatures in eukaryotes. We then hypothesized that high order components could be correlated to gene expression. In result of FPCA analysis for all 27 chromatin marks, we observed that the variation in methylation of residues in histone H3 was systematically better captured by the functional components than the variation in other modifications, while acetylation in H4 was placed in the middle, and variation in acetylation in H2A and H2B, known to have higher rate of histone exchange (or turnover), were generally worst captured, except for H2BK5ac (70 %, 10 components). Considering first component (*ξ*_1_), proportions of variation were in the range 22.2 % (H3K23ac/me2)-65 % (H3K27me3) for pc, and 16 % (H2A.Z)-67.5 % (H3K27me3) for nc (Fig. 4a). Variation in histone variant H2A.Z was poorly recovered for nc genes.

We then computed Pearson correlation coefficients between the scores and the gene expression values (in RPKM) obtained by RNA-seq profiling in H1 hESCs [2] (Fig. 4b). We observed many significant correlations (*P*≤1×10^−6^, correlation test) for up to 5 components, and also that non primary variability (information on higher components) was more relevant for pc genes than for nc genes (Fig. 4b). Thus, either chromatin marks are more involved in pc gene regulation, or many nc genes are not true genes but missannotated transcripts. For pc genes, we found more positive than negative correlations between marks at the first component, and comparable numbers in the second component, generally decreasing for consecutive components of higher order, as expected. However, not always the maximum of correlation (in absolute terms) was located at the first component. Levels of intronic expression in 18410 pc genes [2] correlated most positively with H3K36me3 (*r*=0.1), and most negatively with H3K27me3 (*r*=−0.02), and these were significant only for *ξ*_1_ (data not shown).

These results strongly suggest that higher order principal components of chromatin marks are features that, as breadth or shape of read enrichment [25, 26], have a direct impact on gene expression.

#### Effects of H3K4me3 and H3K9ac correlated variability on gene expression

We then asked if novel insights can also be obtained even when both traditional and FPC-based correlation measures are coincident. As already mentioned, we detected high correlation of the scores between H3K4me3 and H3K9ac ($r_{\eta _{1},\eta _{1}}=0.87$; Fig. 3; Additional file 2). This is in agreement with the view of both marks as associated with active promoters and active regulatory regions, respectively. The maximum FPC-based correlation was almost equal to the ordinary correlation (*r*=0.853 restricted to TSS regions, *r*=0.796 overall). Out of the 19762 regions used in the analysis, 86 % and 96 % of them overlap with significant broad domains on enrichment for H3K4me3/H3K9ac ChIP-seq. Interestingly, we found negative correlation between scores of the second components ($r_{\eta _{2},\eta _{2}}=-0.75$, *P*≤2.2×10^−16^), component that was the only with different shape in the comparison between *ξ*_1_,…,*ξ*_5_ (Additional file 2). Correlations for *ξ*_3_ was also high, but positive ($r_{\eta _{3},\eta _{3}}=0.71$, *P*≤2.2×10^−16^). To study the relation of these correlations with gene expression, we selected TSSs in score quantiles Q1 and Q10, and found that differences in gene expression of corresponding genes between Q1 and Q10 were very significant for *ξ*_2_ (*P*≤2.2×10^−16^, paired t-test), as opposed to *ξ*_1_ (*P*=0.916 for Q1, *P*=0.226 for Q10, paired t-test). This illustrates how relations between eigenfunctions that define chromatin marks are related to transcriptional expression, and how patterns that define the identity of two chromatin marks can be unmasked.

## Conclusions

We have explored variability decomposed into functional components to reveal differences between chromatin modifications at transcription start sites. The biological interpretation of curve information in NGS is relevant, as we and others have shown recently for ChIP-seq peak calling [26, 54–56], RNA-seq [57], and DNA methylation by bisulfite sequencing [58]. The methodology we have proposed and illustrated here continues the progress in the interpretation of next generation sequencing data. Interesting areas to explore in the future are the use of other functional data analysis techniques, such as functional linear regression or functional canonical correlation analysis, and the incorporation of smoothing penalties in the analyses.

In summary, we have shown that curve information in NGS datasets can be well exploited using a functional data analysis approach based on the Karhunen-Loève transform to uncover principal components in the data. We have interpreted the eigenfunctions in epigenomic datasets, and found significant correlations with gene expression. We have shown how this methodology can outperform ordinary correlation measurements to uncover correlations between histone marks that are similarly regulated but which do not directly co-localize. Due to the large amounts of data being generated in this field, we anticipate this dimensionality reduction approach could be useful for large scale exploratory analyses of variation aiming to study genomic and epigenomic maps, as well as their interplay with the rules that govern the histone code.

## Availability and requirements

R scripts are available at: http://github.com/pmb59/KLTepigenome. For one dataset the results were run on 1 CPU with <16GB RAM in ≈2h for pc genes, and ≈3h for nc genes.

## Additional files

Additional file 1
**Roadmap Epigenomics Consortium ChIP-seq data sets used, and results shown in Fig.** 3 **.**

Additional file 2 **Figure S1.** Negative correlation between H3K4me3 and H3K9ac second principal component scores is linked to opposed effects in gene expression. (a) FPCA analysis for H3K4me3 and H3K9ac. First five components are shown. Proportion of variance explained by each eigenfunction is indicated. (b) Scatterplot of the scores for the two chormatin marks. Blue and green lines indicate the first quantile (Q1) for H3K9ac and H3K4me3, respectively. (c) Boxplot of expression values (in RPKM) for protein-coding genes in Q1 and Q10 (outliers are not drawn).

## References

[1] Bernstein BE, Birney E, Dunham I, Green ED, Gunter C, Snyder M (2012). An integrated encyclopedia of DNA elements in the human genome. Nature.

[2] Kundaje A, Meuleman W, Ernst J, Bilenky M, Yen A, Roadmap Epigenomics Consortium (2015). Integrative analysis of 111 reference human epigenomes. Nature.

[3] Satterlee JS, Schubeler D, Ng HH (2010). Tackling the epigenome: challenges and opportunities for collaboration. Nat Biotechnol.

[4] Meyer CA, Liu XS (2014). Identifying and mitigating bias in next-generation sequencing methods for chromatin biology. Nat Rev Genet..

[5] Johnson DS, Mortazavi A, Myers RM, Wold B (2007). Genome-wide mapping of in vivo protein-DNA interactions. Science.

[6] Robertson G, Hirst M, Bainbridge M, Bilenky M, Zhao Y, Zeng T (2007). Genome-wide profiles of STAT1 DNA association using chromatin immunoprecipitation and massively parallel sequencing. Nat Methods.

[7] Song L, Crawford GE (2010). DNase-seq: a high-resolution technique for mapping active gene regulatory elements across the genome from mammalian cells. Cold Spring Harb Protoc.

[8] Gaulton KJ, Nammo T, Pasquali L, Simon JM, Giresi PG, Fogarty MP (2010). A map of open chromatin in human pancreatic islets. Nat Genet.

[9] Rhee HS, Pugh BF (2011). Comprehensive genome-wide protein-DNA interactions detected at single-nucleotide resolution. Cell.

[10] He Q, Johnston J, Zeitlinger J (2015). ChIP-nexus enables improved detection of in vivo transcription factor binding footprints. Nat Biotechnol..

[11] Buenrostro JD, Giresi PG, Zaba LC, Chang HY, Greenleaf WJ (2013). Transposition of native chromatin for fast and sensitive epigenomic profiling of open chromatin, DNA-binding proteins and nucleosome position. Nat Methods.

[12] van Dijk EL, Auger H, Jaszczyszyn Y, Thermes C (2014). Ten years of next-generation sequencing technology. Trends Genet.

[13] McPherson JD (2014). A defining decade in DNA sequencing. Nat Methods.

[14] Risca VI, Greenleaf WJ. Unraveling the 3D genome: genomics tools for multiscale exploration. Trends Genet. 2015. doi: dx.doi.org/10.1016/j.tig.2015.03.010. 10.1016/j.tig.2015.03.010 PMC4490074 25887733

[15] Lee JS, Smith E, Shilatifard A (2010). The language of histone crosstalk. Cell.

[16] Campos EI, Reinberg D (2009). Histones: annotating chromatin. Annu Rev Genet.

[17] de Pretis S, Pelizzola M (2014). Computational and experimental methods to decipher the epigenetic code. Front Genet.

[18] Portela A, Esteller M (2010). Epigenetic modifications and human disease. Nat Biotechnol.

[19] van Dijk EL, Jaszczyszyn Y, Thermes C (2014). Library preparation methods for next-generation sequencing: tone down the bias. Exp Cell Res.

[20] Leek JT, Johnson WE, Parker HS, Jaffe AE, Storey JD (2012). The sva package for removing batch effects and other unwanted variation in high-throughput experiments. Bioinformatics.

[21] Schwartz S, Oren R, Ast G (2011). Detection and removal of biases in the analysis of next-generation sequencing reads. PLoS ONE.

[22] Macaulay IC, Voet T (2014). Single cell genomics: advances and future perspectives. PLoS Genet.

[23] Milosavljevic A (2011). Emerging patterns of epigenomic variation. Trends Genet.

[24] Ernst J, Kellis M (2015). Large-scale imputation of epigenomic datasets for systematic annotation of diverse human tissues. Nat Biotechnol..

[25] Benayoun BA, Pollina EA, Ucar D, Mahmoudi S, Karra K, Wong ED (2014). H3K4me3 breadth is linked to cell identity and transcriptional consistency. Cell.

[26] Schweikert G, Cseke B, Clouaire T, Bird A, Sanguinetti G (2013). MMDiff: quantitative testing for shape changes in ChIP-Seq data sets. BMC Genomics.

[27] Wu T, Liu Y, Wen D, Tseng Z, Tahmasian M, Zhong M (2014). Histone Variant H2A.X deposition pattern serves as a functional epigenetic mark for distinguishing the developmental potentials of iPSCs. Cell Stem Cell.

[28] Hawkins RD, Hon GC, Ren B (2010). Next-generation genomics: an integrative approach. Nat Rev Genet.

[29] Almouzni G, Altucci L, Amati B, Ashley N, Baulcombe D, Beaujean N (2014). Relationship between genome and epigenome - challenges and requirements for future research. BMC Genomics.

[30] Strahl BD, Allis CD (2000). The language of covalent histone modifications. Nature.

[31] Ernst J, Kellis M (2012). ChromHMM: automating chromatin-state discovery and characterization. Nat Methods.

[32] Ernst J, Kellis M (2010). Discovery and characterization of chromatin states for systematic annotation of the human genome. Nat Biotechnol.

[33] Hoffman MM, Buske OJ, Wang J, Weng Z, Bilmes JA, Noble WS (2012). Unsupervised pattern discovery in human chromatin structure through genomic segmentation. Nat Methods.

[34] Hoffman MM, Ernst J, Wilder SP, Kundaje A, Harris RS, Libbrecht M (2013). Integrative annotation of chromatin elements from ENCODE data. Nucleic Acids Res.

[35] Hon G, Ren B, Wang W (2008). ChromaSig: a probabilistic approach to finding common chromatin signatures in the human genome. PLoS Comput Biol.

[36] MacArthur S, Li XY, Li J, Brown JB, Chu HC, Zeng L (2009). Developmental roles of 21 Drosophila transcription factors are determined by quantitative differences in binding to an overlapping set of thousands of genomic regions. Genome Biol.

[37] Pajoro A, Madrigal P, Muino JM, Matus JT, Jin J, Mecchia MA (2014). Dynamics of chromatin accessibility and gene regulation by MADS-domain transcription factors in flower development. Genome Biol.

[38] Zhou J, Troyanskaya OG (2014). Global quantitative modeling of chromatin factor interactions. PLoS Comput Biol.

[39] Lasserre J, Chung HR, Vingron M (2013). Finding associations among histone modifications using sparse partial correlation networks. PLoS Comput Biol.

[40] Assenov Y, Muller F, Lutsik P, Walter J, Lengauer T, Bock C (2014). Comprehensive analysis of DNA methylation data with RnBeads. Nat Methods.

[41] Bock C (2012). Analysing and interpreting DNA methylation data. Nat Rev Genet.

[42] Filion GJ, van Bemmel JG, Braunschweig U, Talhout W, Kind J, Ward LD (2010). Systematic protein location mapping reveals five principal chromatin types in Drosophila cells. Cell.

[43] Julienne H, Zoufir A, Audit B, Arneodo A (2013). Human genome replication proceeds through four chromatin states. PLoS Comput Biol.

[44] Frøslie KF, Røislien J, Qvigstad E, Godang K, Bollerslev J, Voldner N (2013). Shape information from glucose curves: functional data analysis compared with traditional summary measures. BMC Med Res Methodol.

[45] Xu J, Shao Z, Glass K, Bauer DE, Pinello L, Van Handel B (2012). Combinatorial assembly of developmental stage-specific enhancers controls gene expression programs during human erythropoiesis. Dev Cell.

[46] Bernstein BE, Stamatoyannopoulos JA, Costello JF, Ren B, Milosavljevic A, Meissner A (2010). The NIH Roadmap Epigenomics Mapping Consortium. Nat Biotechnol.

[47] Ramsay JO, Silverman BW (2005). Functional Data Analysis.

[48] Ferraty F, Romain Y (2011). The Oxford Handbook of Functional Data Analysis.

[49] Ullah S, Finch CF (2013). Applications of functional data analysis: A systematic review. BMC Med Res Methodol.

[50] Aguilera A, Aguilera-Morillo MC, Escabias M, Valderrama M, Ferraty F (2011). Penalized Spline Approaches for Functional Principal Component Logit Regression. Recent Advances in Functional Data Analysis and Related Topics.

[51] Wang L, Wang S, Li W (2012). RSeQC: quality control of RNA-seq experiments. Bioinformatics.

[52] Carroll TS, Liang Z, Salama R, Stark R, de Santiago I (2014). Impact of artifact removal on ChIP quality metrics in ChIP-seq and ChIP-exo data. Front Genet.

[53] Bailey T, Krajewski P, Ladunga I, Lefebvre C, Li Q, Liu T (2013). Practical guidelines for the comprehensive analysis of ChIP-seq data. PLoS Comput Biol.

[54] Wu H, Ji H (2014). PolyaPeak: detecting transcription factor binding sites from ChIP-seq using peak shape information. PLoS ONE.

[55] Mendoza-Parra MA, Nowicka M, Van Gool W, Gronemeyer H (2013). Characterising ChIP-seq binding patterns by model-based peak shape deconvolution. BMC Genomics.

[56] Mateos J, Madrigal P, Tsuda K, Rawat V, Richter R, Romera-Branchat M (2015). Combinatorial activities of short vegetative phase and flowering locus C define distinct modes of flowering regulation in Arabidopsis. Genome Biol.

[57] Okoniewski MJ, Leśniewska A, Szabelska A, Zyprych-Walczak J, Ryan M, Wachtel M (2012). Preferred analysis methods for single genomic regions in RNA sequencing revealed by processing the shape of coverage. Nucleic Acids Res..

[58] Mayo TR, Schweikert G, Sanguinetti G (2015). M3D: a kernel-based test for spatially correlated changes in methylation profiles. Bioinformatics.

