# Supplementary figures and images for: Uncovering correlated variability in epigenomic datasets using the Karhunen-Loeve transform
Source: BioData Min. 2015 Jul 1;8:20. doi: 10.1186/s13040-015-0051-7 (PMC4488123; doi:10.1186/s13040-015-0051-7)

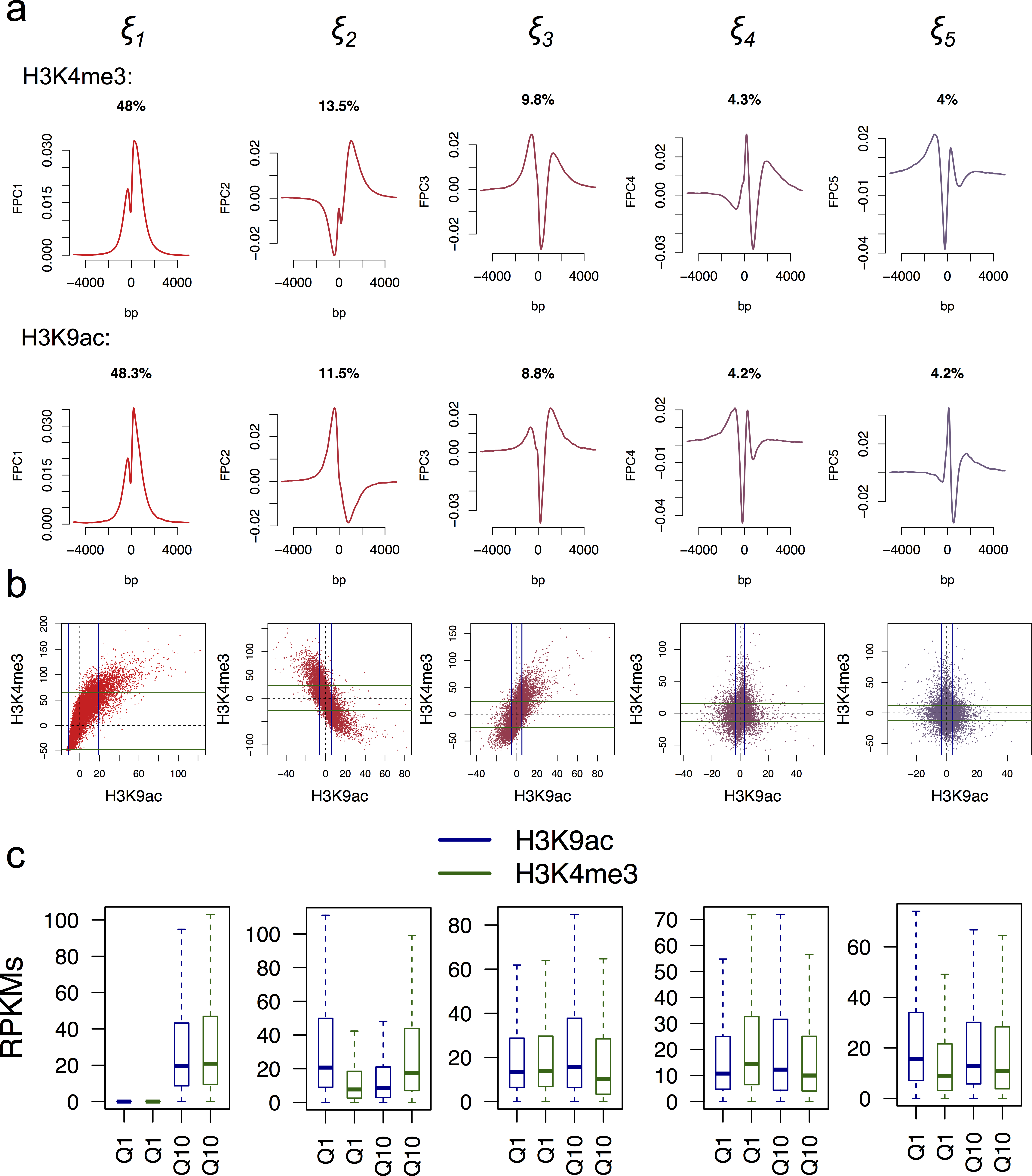

Supplement: Additional file 2 — Figure S1. Negative correlation between H3K4me3 and H3K9ac second principal component scores is linked to opposed effects in gene expression. (a) FPCA analysis for H3K4me3 and H3K9ac. First five components are shown. Proportion of variance explained by each eigenfunction is indicated. (b) Scatterplot of the scores for the two chormatin marks. Blue and green lines indicate the first quantile (Q1) for H3K9ac and H3K4me3, respectively. (c) Boxplot of expression values (in RPKM) for protein-coding genes in Q1 and Q10 (outliers are not drawn). [file 13040_2015_51_MOESM2_ESM.tiff]
